# Three new species of *Carychium* O.F. Müller, 1773 from the Southeastern USA, Belize and Panama are described using computer tomography (CT) (Eupulmonata, Ellobioidea, Carychiidae)

**DOI:** 10.3897/zookeys.675.12453

**Published:** 2017-05-22

**Authors:** Adrienne Jochum, Alexander M. Weigand, Estee Bochud, Thomas Inäbnit, Dorian D. Dörge, Bernhard Ruthensteiner, Adrien Favre, Gunhild Martels, Marian Kampschulte

**Affiliations:** 1 Naturhistorisches Museum der Burgergemeinde Bern, CH-3005 Bern, Switzerland; 2 Institute of Ecology and Evolution, University of Bern, 3012 Bern, Switzerland; 3 Department of Biology, Aquatic Ecosystem Research, University of Duisburg-Essen, 45141 Essen, Germany; 4 Naturhistorisches Museum der Burgergemeinde Bern, CH-3005 Bern, Switzerland; 5 Institute for Ecology, Evolution and Diversity; Senckenberg Biodiversity and Climate Research Center (BiK-F); Senckenberg Gesellschaft für Naturforschung (SGN), Goethe-University (GU), 60438 Frankfurt/M., Germany; 6 Zoologische Staatssammlung München, 81247 München, Germany; 7 Department of Molecular Evolution and Plant Systematics & Herbarium (LZ), Institute of Biology, Leipzig University, 04103 Leipzig, Germany; 8 Department of Experimental Radiology, Justus-Liebig University Giessen, Biomedical Research Center Seltersberg (BFS), 35392 Giessen, Germany; 9 Department of Radiology, Universitätsklinikum Giessen und Marburg GmbH-Standort Giessen, Center for Radiology, 35385 Giessen, Germany

**Keywords:** Computer tomography, conservation, ecology, leaf litter-dwelling species, microgastropods, shell variability

## Abstract

Three new species of the genus *Carychium* O.F. Müller, 1773, *Carychium hardiei* Jochum & Weigand, **sp. n.**, *Carychium belizeense* Jochum & Weigand, **sp. n.** and *Carychium zarzaae* Jochum & Weigand, **sp. n.** are described from the Southeastern United States, Belize and Panama, respectively. In two consecutive molecular phylogenetic studies of worldwide members of Carychiidae, the North and Central American morphospecies *Carychium mexicanum* Pilsbry, 1891 and *Carychium costaricanum* E. von Martens, 1898 were found to consist of several evolutionary lineages. Although the related lineages were found to be molecularly distinct from the two nominal species, the consequential morphological and taxonomic assessment of these lineages is still lacking. In the present paper, the shells of these uncovered *Carychium* lineages are assessed by comparing them with those of related species, using computer tomography for the first time for this genus. The interior diagnostic characters are emphasized, such as columellar configuration in conjunction with the columellar lamella and their relationship in context of the entire shell. These taxa are morphologically described and formally assigned their own names.

## Introduction

Fourteen species of *Carychium* O.F. Müller, 1773, including two non-native, are known in North and Central America. Their distribution ranges from as far north as northern Ontario, Canada (Forsyth and Oldham 2016) through North America (including Bermuda and Jamaica) and south through Central America to Costa Rica (Pilsbry 1948). On a global scale, this genus of tiny, ephemeral, terrestrial snails spans the Nearctic, Palearctic and Indomalayan biogeographic realms. *Carychium* species inhabit mesic microhabitats in tropical and temperate forests, meadows and riparian zones, where they comprise the decomposer community in the duff layer of ecologically stable environments. *Carychium* are found as far north as 64°N in Sweden (von Proschwitz 2006) to as far south as subequatorial Java (Möllendorf 1898).

In two recent papers, Weigand et al. (2011, 2013) addressed the evolution and molecular phylogeny of the worldwide members of the entire family Carychiidae. The authors inferred Asia as the potential origin for the *Carychium* lineage. Subsequent geographical expansions into Europe as well as into North and Central America led to the formation of monophyletic *Carychium* clades in those regions (with the notable exception of the North American species *Carychium nannodes* Clapp, 1905). Within the North and Central American fauna, all Central American species addressed in Weigand et al. (2013) were revealed monophyletic in origin. For the North American taxa, two molecularly distinct evolutionary lineages (ELs) (C9 & C10) were revealed for the morphospecies *C. mexicanum* Pilsbry, 1891. The authors also considered some specimens to belong to *C. exile mexicanum* Pilsbry, 1891, which were genetically revealed to cluster into two different ELs (C1 & C4), partly together with individuals of *Carychium clappi* Hubricht, 1959 and *C. stygium* Call, 1897 (for C1). The taxonomic treatment in Weigand et al. (2013) for the two taxa, *C. mexicanum* and *C. exile mexicanum* was inappropriate as both named morphospecies referred to the same taxonomic unit introduced by Pilsbry (1891). Hence, specimens morphologically identified as being sensu *C. mexicanum* Pilsbry, 1891 were revealed in four different ELs (C1, C4, C9 and C10). Simultaneously, this finding, and the authors’ awkwardly incorrect taxonomic treatment hereof, demonstrated and even contributed to the confusing taxonomic state of *C. mexicanum* (i.e. Hubricht 1974, 1985). In assessing the holotype of *Carychium mexicanum* Pilsbry (ANSP), Hubricht (1974) stated that the specimen was an immature shell and “is thus, a poor specimen for the description of a species”. Additionally, Hubricht (1974) found that “Other specimens identified as *C. mexicanum* by Pilsbry from Mexico and Guatemala do not appear to be specifically distinct from *C. floridanum*”. In his compilation of species distributions of NE American molluscs based upon data presented in H. A. Pilsbry’s Land Mollusca of North America (North of Mexico) (1948), Hubricht (1985) included *Carychium* from the Southeastern United States, stating that the malacofauna of southern Georgia (type locality of *C. hardiei* sp. n.) is “very poorly known”. In addition, Hubricht (1985) explicitly stated that some of the many forms and varieties comprising subspecific categories of certain mollusc taxa are not listed in his compilation. Since Pilsbry considered *C. exile mexicanum* south of Texas a subspecies and an intergradation of *Carychium exile* stock, these forms were not necessarily listed in Hubricht’s (1985) comprehensive work. Hence, confusion abounded long before molecular taxonomy entered carychiid taxonomy via Weigand et al. (2011, 2013). Although the work of Hubricht (1985) is remarkable in his listing of recorded distributions on a state-by-state basis, these records today leave much room for interpretation of which species was distributed where according to the knowledge at the time. As Weigand et al. (2011, 2013) pointed out, many of these entities are likely part of a species complex or represent yet undescribed candidate species or are products of insufficient taxonomic conclusions based on non-diagnostic characteristics. Moreover, our understanding is enriched by the implementation of new methods of analysis as well as the enhanced degree of interpretation enabled by these in the taxonomic process. Primarily, the authors’ investigations highlight the necessity to resolve the current insufficient taxonomic status of *C. mexicanum* (Burch and Van Devender 1980, Hubricht 1974, 1985, Weigand 2012) as part of this present contribution.

**Figure 1.**
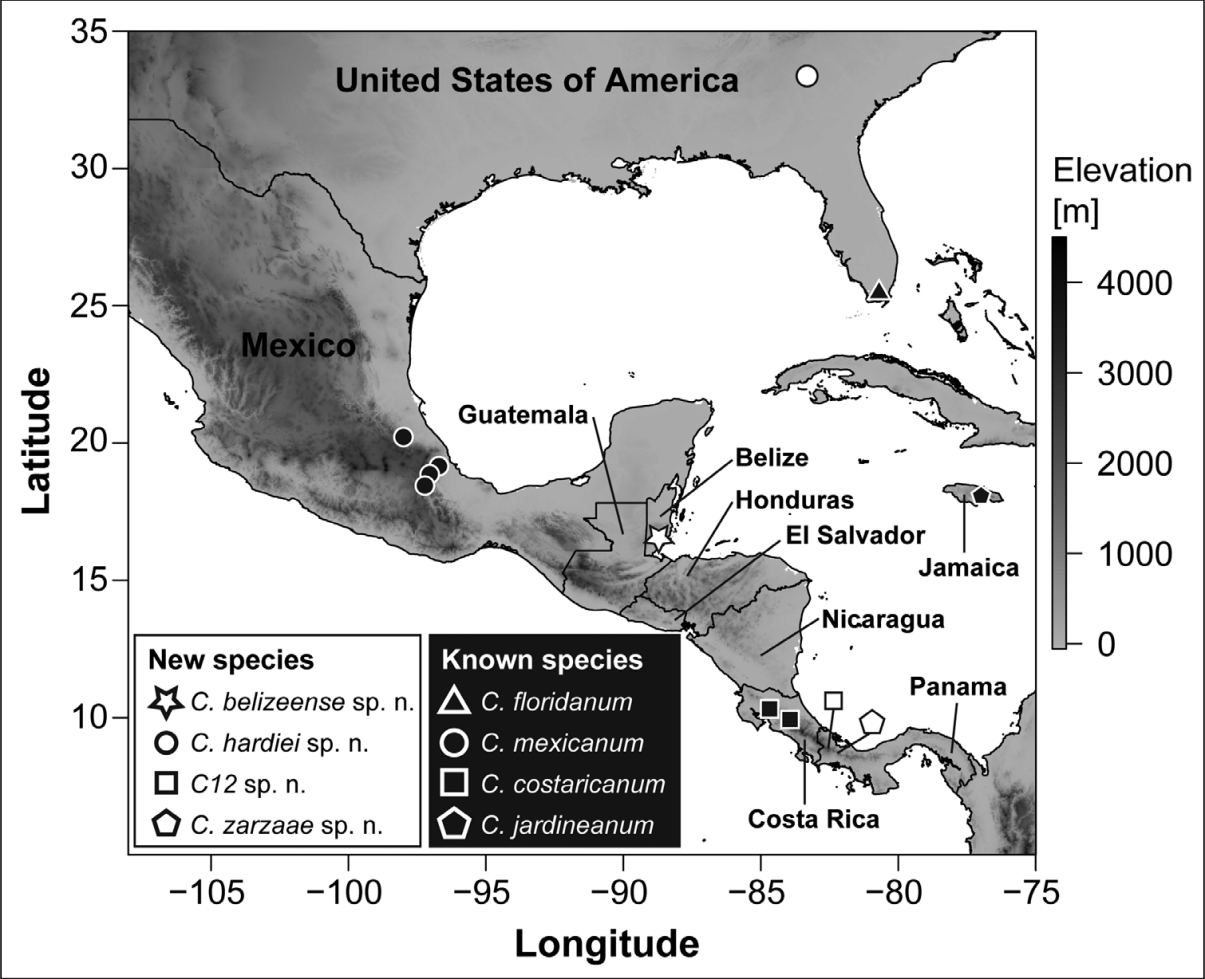
Map indicating geographic position of the new *Carychium* species and type localities of the North American and Central American allied species *C. mexicanum*, *C. costaricanum*, and *C. floridanum*. *Carychium jardineanum* is included in context for Jamaica. The grayscale indicates the local mean elevation. This map was downloaded from WORLDCLIM (Hijmans et al. 2005) and political borders were retrieved from Esri Data and Maps (2002).

Subsequently, Weigand et al. (2013) flagged two additional ELs (C12 and C13) from Panama, which were taxonomically allocated to “*C. mexicanum costaricanum*” in a new combination, following Pilsbry (1948) stating that *C. exiguum costaricanum* sensu E. von Martins, 1898 is probably related to *C. mexicanum* rather than to *C. exiguum* (Say, 1822). Both are also clearly molecularly distinct from topotypic *Carychium costaricanum* E. von Martens, 1898 as well as molecularly distinct from all other carychiid taxa studied thus far. To clarify their position within the bigger picture of the Carychiidae as well as to augment our knowledge of biodiversity in general for North and Central America, these taxa will be described and their shell morphology compared to related taxa from the same region. This concerns the two molecularly flagged and lumped species of *C. mexicanum* lineages C9 and C10 and the Panamanian lineages C12 and C13 (Fig. 1), whereby we describe and provide three of these taxa with a formal name (i.e. C9, C10 and C13). Since the fourth EL (C12) lacks shell material for morphological assessment post molecular investigation and thus, cannot serve as voucher material in a museum collection, this lineage cannot be fully assessed to receive a formal name at this time. The purpose of this present paper is to issue formal taxonomic descriptions to the so far molecularly recognized lineages C9 (former morphospecies *C. mexicanum* USA, Georgia), C10 (former morphospecies *C. mexicanum* Belize) and C13 (former morphospecies *C. mexicanum costaricanum* Panama, Boquete) in Weigand et al. (2013, Fig. 1). Nano-computed tomography (CT) will be used for the first time in *Carychium* to compare interior diagnostic characters such as columellar configuration in conjunction with the columellar lamella and their relationship in context of the entire shell.

## Material and methods

**Figure 2.**
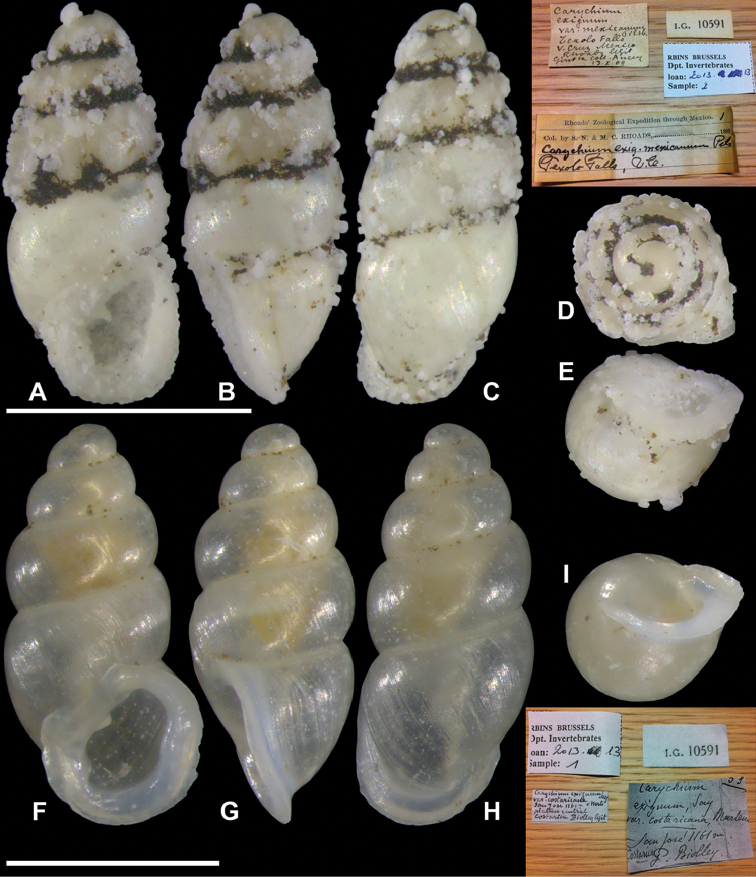
Comparative material, *Carychium mexicanum* Pilsbry, 1891 (RBINS 10591) (ex. Autor) S. N. & M. C. Rhoads Expedition, “Texolo Falls V. Cruz, Mexico Rhoads legit”. **A–E** with Bynes degradation; *Carychium costaricanum* E. von Martens, 1898 (RBINS 10591) original type series **F–I**; **F** peristome thickly callused showing apertural barriers. Scale bar 1 mm.

**Figure 3.**
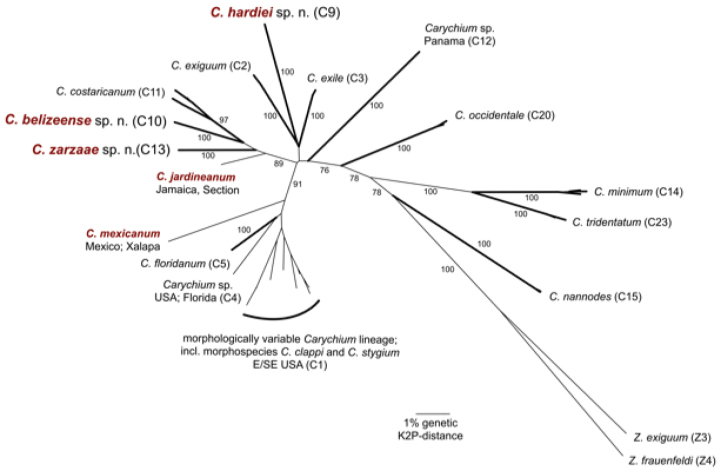
Neighbor-Joining overview topology based on COI K2P-distances for all North and Central American evolutionary lineages accessed in Weigand et al. (2013) and the three newly described *Carychium* species. COI data for *C. mexicanum* and *C. jardineanum* are added. Numbers at the branches indicate bootstrap support for the lineages. BOLD-IDs: *Carychium* lineage C1, E / SE USA: **BARCA014-10**, **BARCA015-10**, **BARCA019-10**, **BARCA020-10**, **BARCA021-10**, **BARCA022-10**, **BARCA025-10**, **BARCA026-10**, **BARCA027-10**, **BARCA028-10**, **BARCA029-10**, **BARCA030-10**, **BARCA127-12**; C2 *C. exiguum* (Say 1822): **BARCA041-10**, **BARCA042-10**, **BARCA043-10**; C3 *C. exile* H.C. Lea, 1842: **BARCA044-10**, **BARCA045-10**, **BARCA046-10**, **BARCA047-10**, **BARCA048-10**, **BARCA049-10**, **BARCA050-10**, **BARCA051-10**; *Carychium* lineage C4, USA; Florida: **BARCA128-12**; C5 *C. floridanum* G.H. Clapp, 1918: **BARCA032-10**, **BARCA033-10**, **BARCA034-10**, **BARCA035-10**, **BARCA037-10**; C9 *C. hardiei* sp. n.: **BARCA038-10**, **BARCA039-10**, **BARCA040-10**; C10 *C. belizeense* sp. n.: **BARCA135-12**, **BARCA136-12**; C11 *C. costaricanum* E. von Martens, 1898: **BARCA137-12**, **BARCA138-12**, **BARCA139-12**, **BARCA140-12**, **BARCA141-12**; *Carychium* lineage C12, Panama: **BARCA142-12**, **BARCA143-12**, **BARCA144-12**; C13 *C. zarzaae* sp. n.: **BARCA145-12**, **BARCA146-12**, **BARCA147-12**, **BARCA148-12**; C14 *C. minimum* O.F. Müller, 1774: **BARCA064-10**, **BARCA065-10**, **BARCA069-10**; C15 *C. nannodes* G.H. Clapp, 1905: **BARCA099-10**, **BARCA100-10**, **BARCA149-12**; C20 *C. occidentale* Pilsbry, 1891: **BARCA054-10**, **BARCA055-10**, **BARCA058-10**, **BARCA059-10**, **BARCA061-10**; C23 *C. tridentatum* (Risso, 1826): **BARCA075-10**, **BARCA079-10**, **BARCA081-10**; *C. mexicanum* Pilsbry, 1891: **BARCA221-17**; *C. jardineanum* (Chitty, 1853): **BARCA222-17**; Z3 *Zospeum exiguum* Kuščer, 1932; **BARCA116-10**; Z4: *Zospeum frauenfeldi* (Freyer, 1855): **BARCA107-10**.

It was not until after the work of Weigand et al. (2013) was published that further *C. mexicanum* material was collected in Puebla (Mexico) ca. 143 km north of its type locality, “Orizaba, Mexico” (Pilsbry, 1891) and in Xalapa, Veracruz (Mexico) ca. 19.6 km distant from Texolo Falls (Cascada de Texolo); these localities were reported by Pilsbry (1948) for *C. mexicanum* from the S. N. Rhoads Expedition (Fig. 2). This subsequent material of *C. mexicanum* is compared here to the evolutionary lineages revealed by Weigand et al. (2013). We provide the CO1 barcode from the newly collected *C. mexicanum* population from Xalapa (BOLD-ID: BARCA221-17) as well as the recently collected *Carychium jardineanum* (Chitty, 1853) from Section, Jamaica (BOLD-ID: BARCA222-17) to include in context with *Carychium* ELs from North and Central America (Fig. 3). The sequences were manually aligned to the existing CO1 alignment using Geneious 5.4.7 (Kearse et al. 2012) with a final length of 607 bp. The Neighbour-Joining overview topology was generated in MEGA 6.06 (Tamura et al. 2013) using 1,000 bootstrap replicates, the Kimura-2-Parameter substitution model generally applied to DNA barcode data and the pairwise deletion option.

*Carychium jardineanum* (Chitty, 1853) was collected by the first author under the permit Ref. Nrs. 18/27 and 18/70 issued by the National Environment and Planning Agency (NEPA), Kingston, Jamaica.

For the species descriptions, shell measurements are expressed as **SH** (shell height), **SD** (shell diameter), **PH** (peristome height) and **PD** (peristome diameter). The number of whorls of each shell was counted according to the method described in Kerney et al. (1979). Localities of the material are presented in Table 1.

**Table 1.** Individuals, locality and museum data.

*Carychium* Species	Locality and collection data	Latitude / Longitude	Museum/ Voucher No.	EL/Coll. No.
*C. belizeense* sp. n.	Belize, Maya Mountains, Bladen Nature Reserve. In leaf litter. 404 m, 1 May 2010, leg. Dan Dourson	16.557167, −88.707833	NMBE 549923–549925, ANSP A24823, SMF 341639, CM 155815, UF 489972	C10
*C. costaricanum* (E. von Martens, 1898)	Costa Rica, Alajuela Prov., Puntarenas, Reserva Biológica Bosque Nuboso Monteverde. Sendero Roble-Chomogo. In moist leaf litter. 18 Feb. 2000. 1650 m, leg. Ira Richling	10.301333, −84.790167	MZUCR251	C11
*C. costaricanum* (E. von Martens, 1898)	“Central Costa Rica: San José (Boilley)”. Original type series	9.935443, −84.101844	RBINS 10591.[1]	
*C. floridanum* (Clapp, 1918)	“Snapper Creek Hammock, No. 8569 of my collection” [= “about 8 miles south of Miami, Fla.”]. Syntype	25.69344, −80.358108	CM 46540	
*C. hardiei* sp. n.	USA, Georgia, Flovilla, Butts County, Indian Springs State Park. Leaf litter in drainage basin. 5 April 2010, leg. Adrienne Jochum	33.242367, −83.92035	NMBE 549920–549922, ANSP 467825, CM 155814, SMF 341638, UF 489973	C9
*C. jardineanum* (Chitty, 1853)	Jamaica, Portland Parish, Blue and John Crow Mountains, Section	18.087917, −76.70535		AJC 2320
*C. mexicanum* (Pilsbry, 1891)	Type locality: “Orizaba, Mexico”. “Hills around Orizaba, at an altitude of about 500 feet above the town.”	18.870374, −97.083377	ANSP 61628 (Lectotype)	
*C. mexicanum* (Pilsbry, 1891)	Mexico, Veracruz, Texolo Falls. Rhoads Exp. “ex Auct” (secondary material)	19.416944, −97.0000	RBINS 10591.[2]	
*C. mexicanum* (Pilsbry, 1891)	Mexico, Puebla, near Texcapa. On moist stick. 30 April 2012. 1310 m, leg. Eugenia Zarza	20.19422, −98.03752		AJC 2092
*C. mexicanum* (Pilsbry, 1891)	Mexico, Veracruz State, Xalapa, Botanical Garden of Instituto de Ecologia. 28 April 2012. 1249 m, leg. Eugenia Zarza	19.513361, −96.940278		AJC 2090
*C. zarzaae* sp. n.	Panama, Chiriqui Prov., Boquete (path near Boquete). On moist stick in leaf litter. 12 Sept. 2011. 1680 m, leg. Eugenia Zarza	8.824767, −82.495833	NMBE 549926–549927, CM 155816	C13; ex AJC 1902; ex AJC 1903
*Carychium* sp. (C12 in Weigand et al. 2013)	Panama, Chiriquí, Sendero el Retoño, El Pila, Parque National la Amistad. On moist stick in leaf litter. 9 Sept. 2011. 2230 m, leg. Eugenia Zarza	8.896611, −82.617861	N/A	C12; ex AJC 1904; ex AJC 1905

Several qualitative aspects of shell morphology are addressed: peristome shape; whorl profile (whorl convexity); regularity of the protoconch; teleoconch sculpture; development of apertural barriers visible in frontal view, including the presence of a deeply immersed denticle/lamella on the parieto-columellar region of the aperture; development of the columellar lamella as discernable in the CT scans of the ventral, dorsal, side-left and side-right perspectives of the adult shell.

Material is housed in the following collections:

**AJC** Adrienne Jochum Collection: formerly Institute of Ecology, Evolution & Diversity, Phylogeny & Systematics Collection, Goethe-Universität, Frankfurt am Main, Germany

**ANSP** Academy of Natural Sciences of Drexel University, Philadelphia, PA, USA

**CM** Carnegie Museum of Natural History, Pittsburgh, PA, USA

**MZUCR** Museo Zoológico de la Universidad de Costa Rica, San José, Costa Rica

**NMBE** Naturhistorisches Museum der Burgergemeinde Bern, Bern, Switzerland

**RBINS** Royal Belgian Institute of Natural Sciences, Brussels, Belgium

**SMF** Forschungsinstitut und Naturmuseum Senckenberg, Frankfurt am Main, Germany

**UF** University of Florida, Florida Museum of Natural History, Gainesville, FL, USA

### Image acquisition

**Micro-CT**: All *Carychium* in this work, except for *Carychium zarzaae* sp. n., were imaged using a nano-computed tomography system (micro-CT), the SkyScan 2011 (Bruker MicroCT, Kontich, Belgium) at the Department of Experimental Radiology, Justus-Liebig University Biomedical Research Center Seltersberg (BFS), Giessen, Germany. The system contains an open pumped type X-ray source, a LaB6 cathode and a transmission anode consisting of a tungsten-coated beryllium window. Enhanced edge sharpness and submicron resolution are gained by a high-focused X-ray spot of <400 nm side length (see Langheinrich et al. (2010) for more details). The *Carychium* were mounted and scanned 185° around their vertical axis in rotation steps of 0.23° at 80 kV tube voltage and 120 μA tube current. Reconstruction was performed using the Feldkamp cone beam reconstruction algorithm. Image resolution was 1.75 μm isotropic voxel side length with a grey scale resolution of 8 bit. Digital image post processing and visualization (maximum intensity projection – MIP, volume compositing and summed voxel projection) were displayed using the ANALYZE software package (ANALYZE 11.0, Mayo Clinic, Rochester, MN, USA). *Carychium zarzaae* sp. n. was imaged at the Zoologische Staatssammlung München, Munich, Germany. Scanning was performed with a Phoenix Nanotom m (GE Measurement & Control, Wunstorf, Germany) cone beam CT scanner at a voltage of 80 kV and a current of 325 mA using a tungsten (“Standard”) target. 1440 projection images were taken during a 360° rotation at a total duration of 96 minutes. Voxel size was 0.826 µm. The 16 bit data set generated by reconstruction was cropped and converted to 8 bit using VGStudio MAX 2.2 software (Volume Graphics, Heidelberg, Germany). Further visualization procedures were carried out with Amira 6.2 software (FEI Visualization Sciences Group, Burlington MA, USA) applying manual segmentation for discrimination of external and internal shell structures. Final visualization was enabled using the Volume Rendering tool.

**Digital images**: Shells were imaged using a Leica DFC425 digital camera attached to a Leica M205 C stereo microscope (Wetzlar, Germany), using IMS Client analysis image system software (Imagic Bildverarbeitung AG, Glattbrugg, Switzerland) for measurements.

For convenience in comparing the images between species descriptions, figures are assigned in geographical context from North America to Central America, starting with *Carychium hardiei* sp. n. (Figs 4–5), then allied species (Figs 6–9), *Carychium belizeense* sp. n. (Figs 11–12), *Carychium zarzaae* sp. n. (Figs 13–14) and lineage C12 (Fig. 15).

## Taxonomy

### Family Carychiidae Jeffreys, 1830

#### Genus *Carychium* O.F. Müller, 1773

##### Carychium hardiei

Taxon classificationAnimaliaEupulmonataCarychiidae

Jochum & Weigand sp. n.

http://zoobank.org/F01FE191-CB7F-429A-AEE8-C0E328230E0C

Figures 4 , 5

Weigand et al., 2013: 3, Fig. 1 37|C9; Seq. ID: BARCA038-10, BARCA039-10, BARCA040-10

###### Material.

Holotype (NMBE 549920/1 ex AJC 1444): USA, Georgia, Flovilla, Butts County, Indian Springs State Park; N 33.242367 W -83.92035; under moist leaf litter in drainage basin; 5 April 2010; leg. Adrienne Jochum.

**Figure 4.**
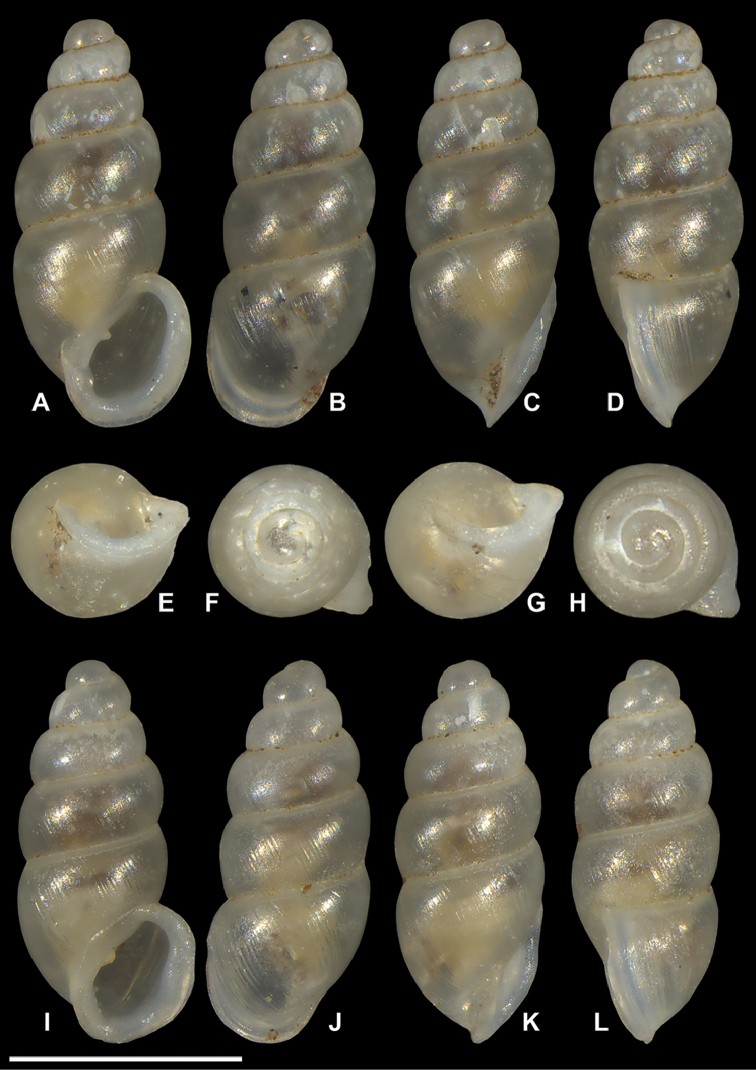
*Carychium hardiei* sp. n. **A**–**F** holotype (NMBE 549920/1) **G–L** paratype shell (NMBE 549921/8). Scale bar 1 mm.

**Figure 5.**
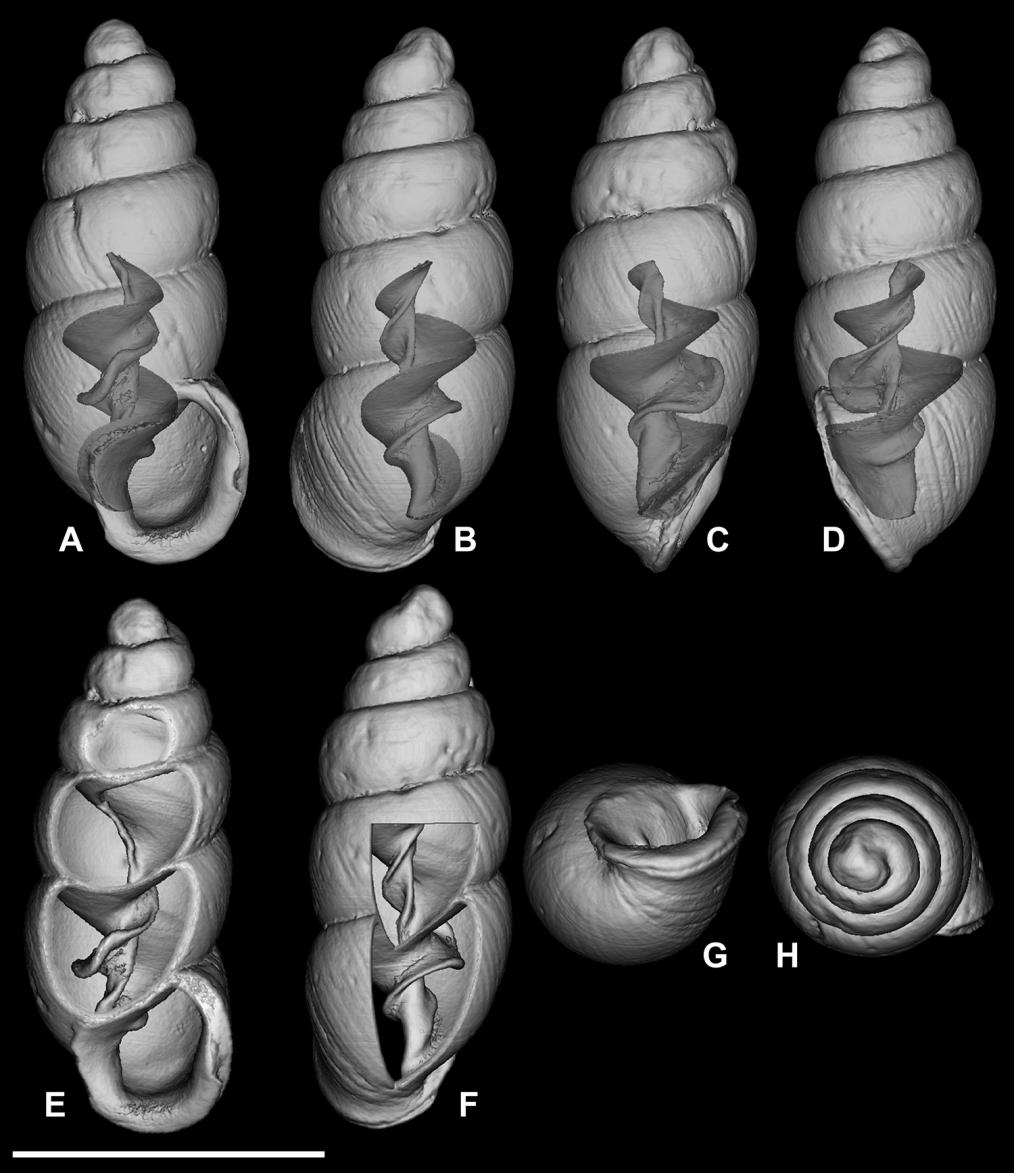
*Carychium hardiei* sp. n. **A–F** CT scans showing columellar apparatus of paratype (NMBE 549921/8) **G** umbilical view **H** aerial view of protoconch and spire. Scale bar 1 mm.

Paratypes: locus typicus 8 shells (NMBE 549921/8 ex AJC 1444); 8 specimens in alcohol (NMBE 549922/8 ex AJC 1444); 8 shells (SMF 341638 ex AJC 1444); 7 shells (ANSP 467825 ex AJC 1444); 7 shells (CM 155814 ex AJC 1444); 6 shells (UF 489973 ex AJC 1444); 24 shells previously preserved in formalin and dried (AJC 1445); data as the holotype.

###### Diagnosis.

Shell ca. 1.75 mm in height, transparent, elongate-pupiform with an entire, elliptical-oblique, moderately thickened peristome, including a small, deeply set parietal denticle and a slight columellar-basal callus.

###### Description

(material from type locality). Measurements of holotype and paratypes are provided in Table 2.

**Table 2.** Measurement of *Carychium* shells in frontal view. Species: *Carychium belizeense* sp. n., N = 6; *Carychium hardiei* sp. n., N = 6; *Carychium floridanum* Clapp, 1918 (CN 46540), N = 2; *Carychium costaricanum* E. von Martens, 1898 (RBINS 10591.[1]), N = 1; *Carychium mexicanum* Pilsbry, 1891 (RBINS 10591.[2], N = 1; *Carychium mexicanum* (AJC 2092), N = 4; *Carychium zarzaae* sp. n., N = 2. Abbreviations: SH – shell height, SD – shell diameter, PH – peristome height, PD – peristome diameter, W – Whorl number. All measurements in millimetres.

*Carychium* species	Museum No./Coll. No.	Sample	SH	SD	PH	PD	W
*C. belizeense* sp. n. Holotype	NMBE 549923	1	1.76	0.76	0.64	0.62	4.75
*C. belizeense* sp. n. Paratype	NMBE 549924/8	2	1.77	0.76	0.58	0.55	4.75
*C. belizeense* sp. n. Paratype	NMBE 549924/8	3	1.83	0.80	0.63	0.57	5.00
*C. belizeense* sp. n. Paratype	NMBE 549924/8	4	1.77	0.76	0.62	0.59	5.00
*C. belizeense* sp. n. Paratype	NMBE 549924/8	5	1.68	0.71	0.56	0.51	5.00
*C. belizeense* sp. n. Paratype	NMBE 549924/8	6	1.70	0.77	0.62	0.59	4.63
**Mean *C. belizeense***			**1.75**	**0.76**	**0.61**	**0.57**	**4.86**
*C. hardiei* sp. n. Holotype	NMBE 549920	1	1.86	0.83	0.70	0.60	4.80
*C. hardiei* sp. n. Paratype	NMBE 549921/8	2	1.74	0.81	0.62	0.57	5.00
*C. hardiei* sp. n. Paratype	NMBE 549921/8	3	1.75	0.78	0.62	0.59	4.80
*C. hardiei* sp. n. Paratype	NMBE 549921/8	4	1.69	0.77	0.60	0.55	5.38
*C. hardiei* sp. n. Paratype	NMBE 549921/8	5	1.74	0.79	0.63	0.57	5.00
*C. hardiei* sp. n. Paratype	NMBE 549921/8	6	1.70	0.75	0.60	0.55	5.00
**Mean *C. hardiei***			**1.75**	**0.79**	**0.63**	**0.57**	**5.00**
*C. floridanum* Clapp, 1918 (CT)	CM 46540	1	1.68	0.76	0.62	0.58	4.75
*C. floridanum* Clapp, 1918 (CT)	CM 46540	2	1.62	0.72	0.59	0.59	4.75
**Mean *C. floridanum***			**1.65**	**0.74**	**0.61**	**0.59**	**4.75**
*C. costaricanum* Von Martens, 1898	RBINS 10591.[1]	1	**1.88**	**0.93**	**0.76**	**0.76**	**4.50**
*C. mexicanum* Pilsbry, 1891	RBINS 10591.[2]	1	1.60	0.70	0.63	0.57	4.00
*C. mexicanum* (topo. vicinity) (CT)	AJC 2092	1	1.64	0.80	0.70	0.58	4.00
*C. mexicanum* (topo. vicinity)	AJC 2092	2	1.63	0.72	0.59	0.54	4.25
*C. mexicanum* (topo. vicinity)	AJC 2092	3	1.68	0.80	0.69	0.59	4.50
*C. mexicanum* (topo. vicinity)	AJC 2092	4	1.66	0.79	0.69	0.58	4.20
**Mean *C. mexicanum***			**1.65**	**0.78**	**0.67**	**0.57**	**4.23**
*C. zarzaae* **sp. n. Holotype**	AJC 1902/NMBE	2	1.74	0.84	0.67	0.55	4.25
*C. zarzaae* sp. n. Paratype (CT)	AJC 1902/NMBE	1	1.69	0.82	0.65	0.59	4.25
**Mean *C. zarzaae***			**1.72**	**0.83**	**0.66**	**0.57**	**4.25**

Shell minute, elongate-pupiform, pellucid when fresh, opaque shiny when older. Aperture elliptical-oblique, somewhat higher than wide, taking up less than one third the shell height, outer lip moderately reflected, thin above, increasingly thickened on its outermost extension by a heavy deposit of callus upon its surface and inner edge; columellar margin has a callus and an acute entering fold above. Whorls convex, moderately rounded, ranging from 4.8–5.4 in number. Suture deep. Protoconch bulbous. Teleoconch sculpture consists of weak, irregular oblique striae. Maximum width of body whorl extends 1/6 beyond the rim of the peristome in side view facing left (Fig. 4D, L). Palatal rim appears alabaster-like. In profile view facing left, the peristome of the mature shell gently curves in at the upper edge, expands moderately and then bends gently back at the base of the shell in alignment with the central axis (Fig. 4D, L). A small, deeply set, parietal denticle is almost centrally positioned on the parietal face (Fig. 4A, I). The tiny, deeply set parietal denticle is partially visible in umbilical view (Fig. 5G). Internally, from the dorsal and aperture-facing-right perspectives, the columella bears a single, simple elongate lamella beginning one third the distance from the top of the penultimate whorl (Fig. 5B–C, F), which elaborates into a characteristic, broadly winged, tongue-like structure in aperture-facing-left position (Fig. 5D). In ventral perspective, the lamella is slightly sinuate, whereby the first thickened flexure directed upward is in alignment with the columellar axis (Fig. 5A). The lamella culminates at the base of the gently twisted columella as a small, deeply set, well-formed, non-sinuous denticle. The base of the columella spindle (portion under the tongue-like lamella) is moderately long and dilated by the final manifestation of the columellar lamella forming the parietal denticle (Fig. 5E–F).

###### Differential diagnosis.

**Figure 6.**
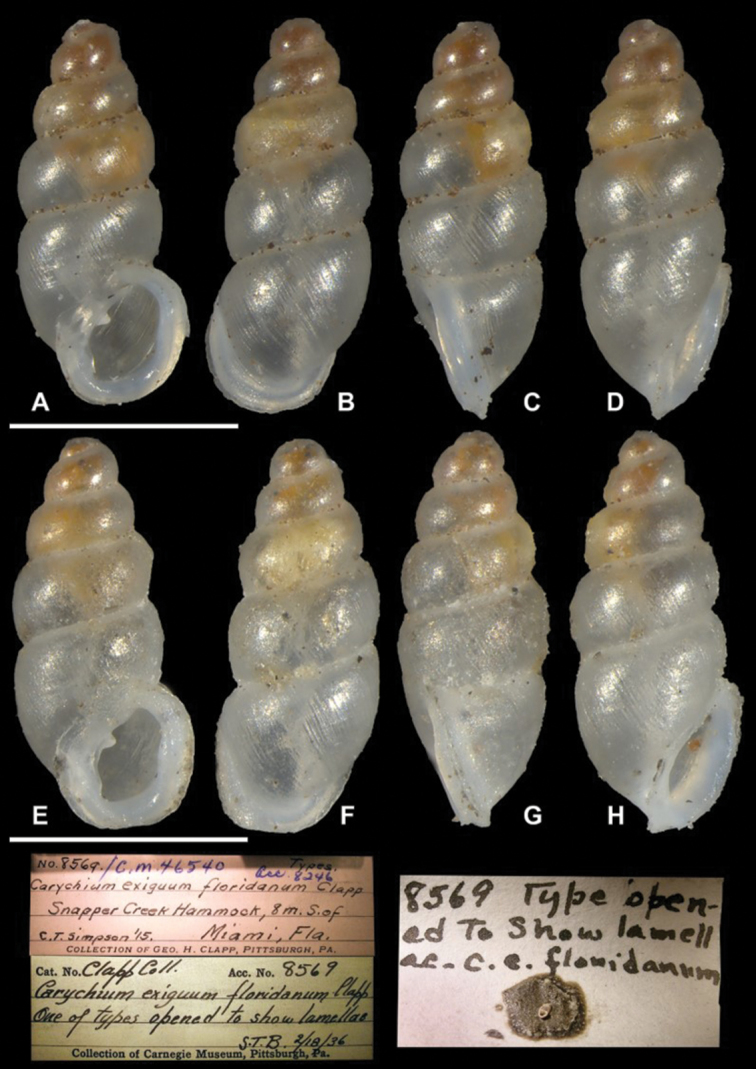
Comparative material, *Carychium floridanum* Clapp, 1918 (CM 46540). **A–H** syntypes and specimen information; **E** peristome with thick parieto-columellar callus and prominent parietal denticle. Scale bar 1 mm.

**Figure 7.**
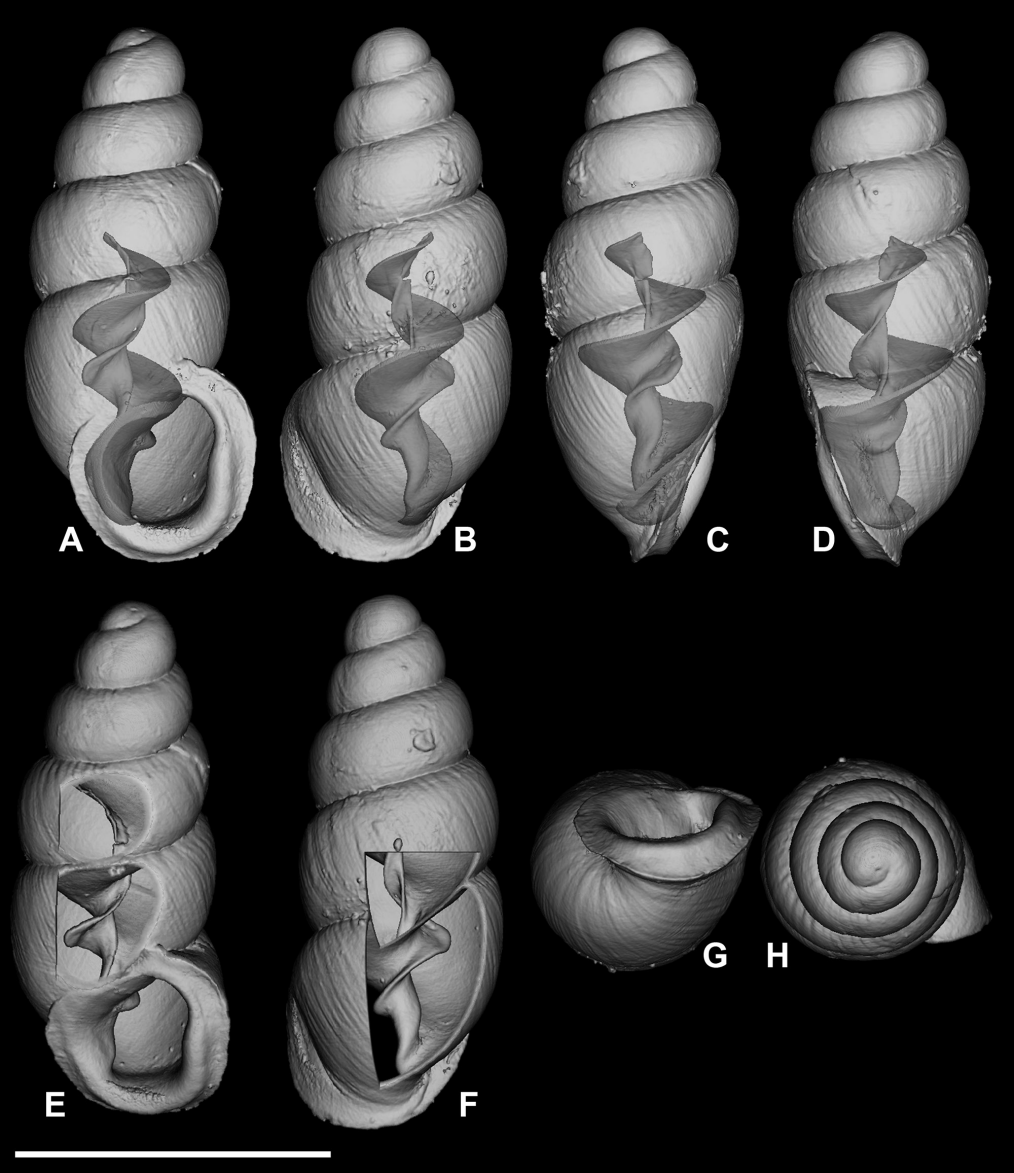
Comparative material, *Carychium floridanum* Clapp, 1918 (CM 46540). **A–F** CT scans showing columellar apparatus and simple lamellar sinuosity of syntype **C** moderately thickened S-shaped lamella **A, E** zones of increased callus concentration on the peristome **G** umbilical view showing turned back rim of peristome. Scale bar 1 mm.

**Figure 8.**
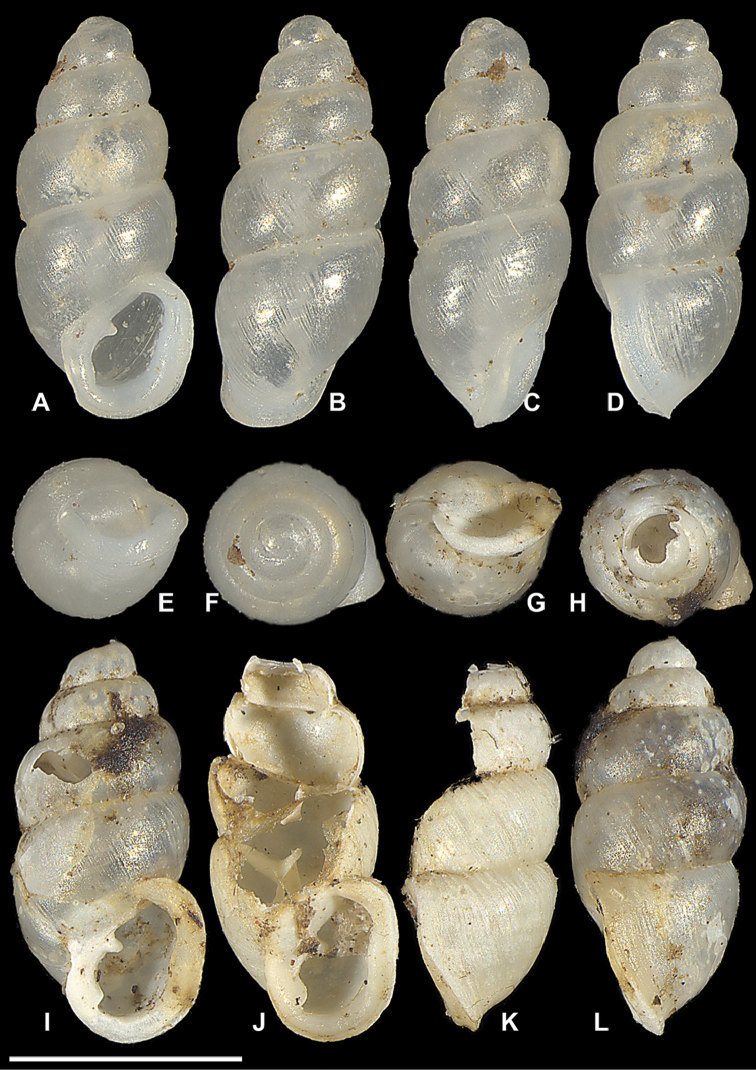
Comparative material, *Carychium mexicanum* Pilsbry, 1891 (AJC 2092) from Puebla, Mexico. **A–L** fresh shells collected proximal to topotypic locality (Orizaba, Mexico) **A, I–J** degrees of variation in apertural barriers. Scale bar 1 mm.

**Figure 9.**
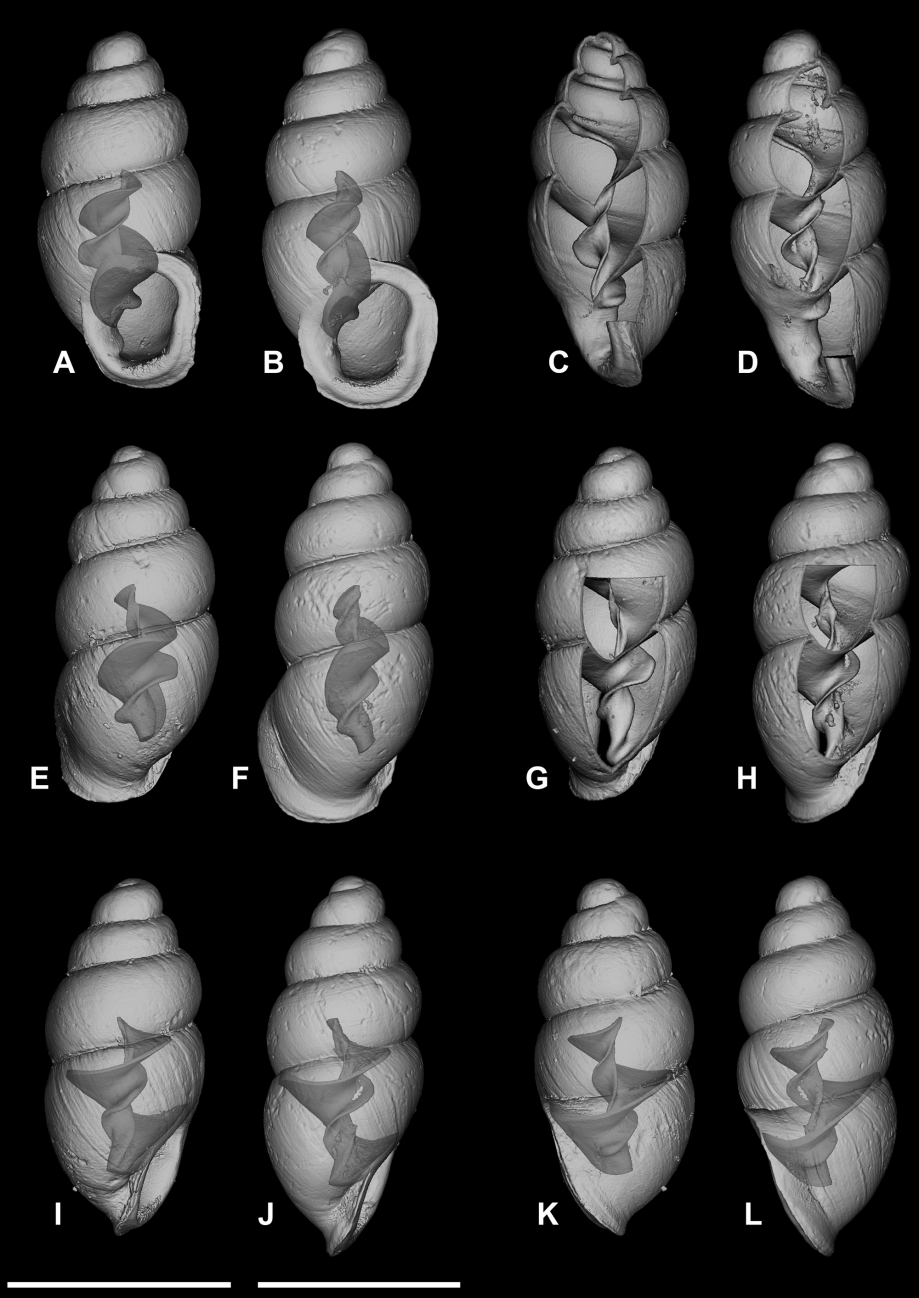
Comparative material, *Carychium mexicanum* Pilsbry, 1891 (AJC 2092) from Puebla, Mexico **A, C, E, G, I, K** and topotypic *C. costaricanum* E. von Martens, 1898 (AJC 2093) **B, D, F, H, J, L**; **A–L** CT scans showing columellar apparatus and configuration of the columellar lamella in both species **A–L**. Scale bar 1 mm.

Differs from *Carychium floridanum* G.H. Clapp, 1918 (Figs 6–7) by the more bulbous protoconch, the thinner, less rounded and less swollen peristome, the enhanced elaboration of sinuosity and thickness of the columellar lamella (interior ventral view) and the downward projection of the lamella in tongue-like form, the presence of the parietal denticle in umbilical view and the reduced size of the deeply-set parietal denticle; from *C. mexicanum* (RBINS 10591.2, “Texolo Falls, V. Cruz [Veracruz], Mexico” (Pilsbry 1948)) (Figs 2A–E, 8–9) by its larger and more tapered shell, the thinner, less swollen peristome, the first flexure of the sinuate lamella synchronal with the shell axis as seen in the interior ventral view, the more elaborate configuration of the columellar lamella (in side view-left perspective) and the tininess of the parietal denticle; in *C. costaricanum* (RBINS 10591.1), “San José Plateau Costa Rica” (E. von Martens 1898) (Figs 2F–I, 9), the shell is more robust with increased roundness of the whorls, the aperture is markedly more auriform, the peristome is greatly thickened (swollen) and rounder with a thick columellar-basal callus in the inner left-hand corner, the columellar lamella is simple and smooth without wing-like elaboration, the parietal denticle is thick and less finely defined as in *C. hardiei*.

DNA barcode data can clearly delineate *Carychium hardiei* sp. n. from all other North and Central American taxa, demonstrating its lowest K2P genetic distance of 5.4 % to *C. exile* (Fig. 3). For comparison, the genetic distances between individuals of the two widely distributed European species *Carychium minimum* O. F. Müller, 1774 and *Carychium tridentatum* (Risso, 1826), for which the most sophisticated DNA barcode dataset exists (with more than 270,000 pairwise estimates), are always ≥ 4.3 %, and within-species K2P genetic distances not above 3.4 % (Weigand et al. 2014).

###### Etymology.

The new species is named after the American chemical engineer, naturalist, field biologist and long-time friend of the first author, Frank Hardie of South Carolina. Frank’s tireless hours in the field contributed substantially to the *Carychium* dataset encompassed in Weigand et al. (2011, 2013) as well as to the burgeoning dataset of our ongoing investigation of the Carychiidae. Without his dedication and perseverance in the field, the genus *Carychium* would be remarkably poorer for it. In accordance with Mr. Hardie’s heritage in the southeastern USA, we gratefully dedicate *Carychium hardiei* from the American southeastern State of Georgia in his name.

###### Distribution.

**Figure 10.**
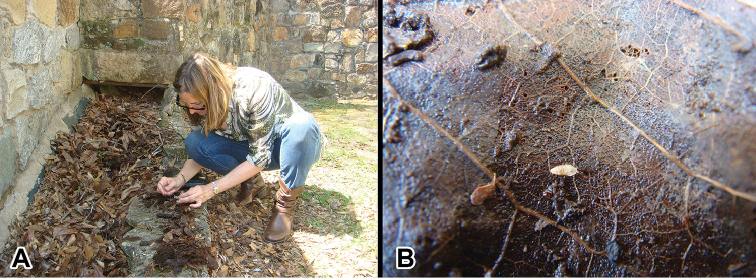
Type locality of *Carychium hardiei* sp. n., Indian Springs State Park, Flovilla, Georgia, USA. **A** collector A. Jochum; **B** *Carychium hardiei* in action on rotten leaf.

This species is only known from the type locality at Indian Springs State Park, ca. 90 km south of Atlanta, Georgia. The drainage basin where these individuals were collected was located proximate to the park entrance and adjacent to the latrine complex (Fig. 10). It remains questionable whether any of the so-called *C. mexicanum* distributions included in Hubricht (1985) for the southeastern United States include *C. hardiei* sp. n. Moreover, Hubricht (1985) explicitly states that faunistic surveys were largely lacking in the State of Georgia and thus, the existence of *C. hardiei* sp. n. could well have been overlooked completely.

###### Ecology.

Mixed deciduous leaf litter.

###### Conservation.

In the drainage basin where this species was found (see above), live individuals occurred in relative abundance, suggesting that *C. hardiei* has optimum ecological conditions to survive there. Still, on a global scale, its current distribution may be limited to the 2.14 square kilometres of woodland in the middle of Georgia, regarded as Indian Springs since 1825 and as a “State Forest Park” since 1927. In conjunction with the Guidelines for the IUCN Red List (IUCN Standards and petitions Subcommittee 2014) it likely constitutes a Critically Endangered narrow range endemic (CR B1). Habitat disturbance by pollution and human encroachment via tourism or urban development may pose the greatest threat.

###### Remarks.

In a subsequent study of the degrees of morphological variation observed in Carychium exiguum var. mexicanum Pilsbry, 1891, Pilsbry (1948) introduced the description “*Carychium exile mexicanum*” to make a point while comparing the variations of *Carychium exile* H.C. Lea, 1842 forms collected in Texas with those from the southern extension of the Carychium exiguum var. mexicanum range. This temporary and awkward nomenclatural construction was subsequently used by Weigand et al. (2013) when these authors assessed their material, overlooking Pilsbry’s (1948) momentarily useful, but erroneous nomenclatural construction in naming the respective morphospecies for the evolutionary lineages C1 and C4 from northern Georgia, Alabama and northern Florida. Regarding *C. hardiei* sp. n. and our investigations of *C. mexicanum* here, Pilsbry’s (1948) considerations were very insightful and are still relevant considering his broader observation of southern forms (diversity) in what we now see, using cutting-edge methods, as taxonomically unrecognized species. For Carychium exiguum var. mexicanum, Pilsbry (1948) concluded “It is the tropical representative of the *exile* stock and probably to be considered a southern subspecies of that, intergrading with it in Texas.”

Since Pilsbry’s (1891) initial description of “Carychium exiguum var. mexicanum” from “Orizaba, Mexico”, the distribution of *Carychium mexicanum* has up to now, spanned across the southern United States from Georgia and Florida west to Texas (Hubricht 1985), and south to Guatemala (Thompson 2011). However, in context of Weigand et al.’s (2013) recent molecular investigation (based on COI, 16S and H3) and our use of CT scans to assess shell morphology here, we are clearly dealing with separate species. Moreover, this work not only corroborates Pilsbry’s astute morphological observations, but also, shows that what he considered as subspecies were indeed different entities we now consider separate lineages quite distinct from the “true” *C. mexicanum* collected in Mexico.

##### Carychium belizeense

Taxon classificationAnimaliaEupulmonataCarychiidae

Jochum & Weigand sp. n.

http://zoobank.org/35C8EC6B-CF0C-4FA4-A85C-BFC788C5E047

Figures 11 , 12

Weigand et al., 2013: 3, Fig. 1 40|C10; Seq. ID: BARCA BARCA135-12; BARCA136-12.

###### Material.

Holotype (NMBE 549923/1 ex AJC 1588): Belize, Maya Mountains, Toledo District, Forest Hill, Bladen Nature Reserve; N16.557167, W-88.707833, alt. c. 404 m, in wet depressions between layers of moist leaves; 12 July 2012; leg. Dan Dourson.

**Figure 11.**
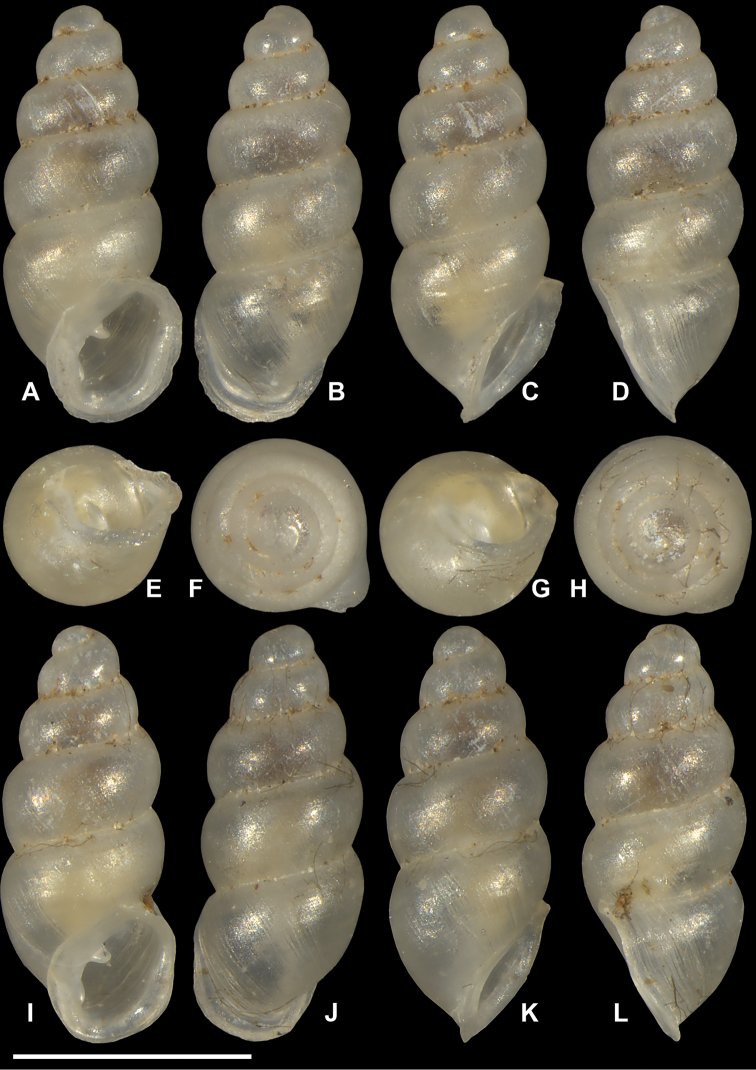
*Carychium belizeense* sp. n. **A–F** holotype (NMBE 549923/1) **G–L** paratype shell (NMBE 549924/8) **A, I** prominent parietal denticle and parieto-columellar callus. Scale bar 1 mm.

**Figure 12.**
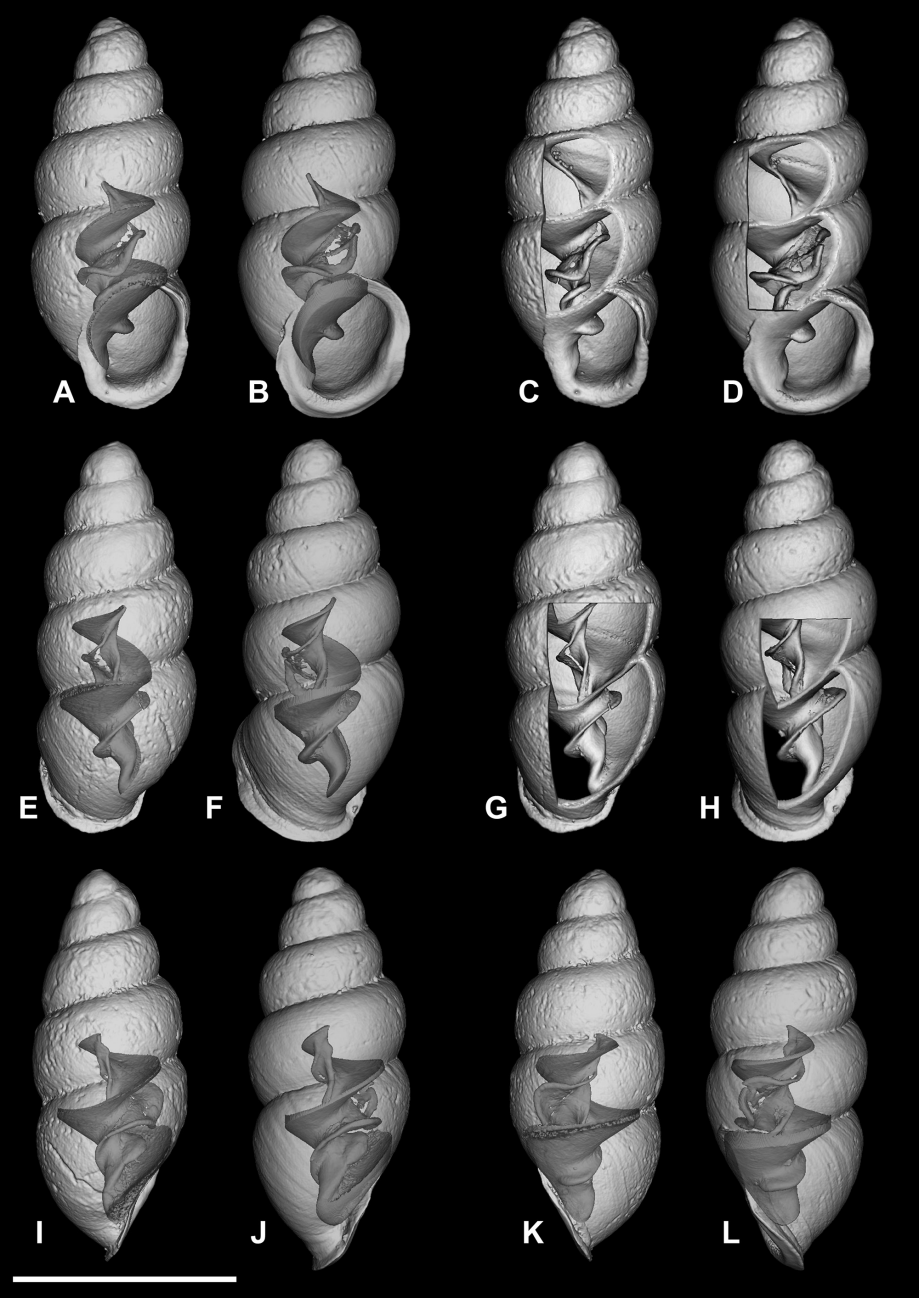
*Carychium belizeense* sp. n. **A–L** CT scans showing columellar apparatus of paratype (NMBE 549924/8) **C–D** two-tiered, tightly coiled columellar lamella and prominent parietal denticle **I–J** highly sinuate tongue-like primary lamella tightly arching over swollen secondary lamella. Scale bar 1 mm.

Paratypes: locus typicus: 8 shells (NMBE 549924/8 ex AJC 1588) (2 CT-scanned) and 5 specimens in alcohol (NMBE 549925/5 ex AJC 1589); 5 shells (SMF 341639 ex AJC 1589); 4 shells (UF 489972 ex AJC 1589); 4 shells (CM 155815 ex AJC 1589); 6 specimens in alcohol (ANSP A24823 ex AJC 1588); data as the holotype.

###### Diagnosis.

Shell c. 1.75 mm in height, elongate-pupiform with an elliptical-shaped aperture, pellucid, thick peristome with a columellar-basal apertural barrier and a pronounced parietal denticle. Although it bears one more whorl than *C. costaricanum*, it is smaller than this species but larger than *C. mexicanum*. Internally, *C. belizeense* has a highly sinuate, tightly coiled, two-tiered columellar configuration.

###### Description.

Measurements are provided in Table 2. Shell minute, elongate-pupiform, transparent when fresh, robust, with about 4–5 highly convex whorls and a deeply incised suture; with irregular striations or blunt growth lines on the body whorl. Protoconch bulbous. Teleoconch is irregularly smooth with course texture and vague indentations disrupting the overall glossy sheen. Peristome elliptical and thick, only slightly higher than wide, closely adhering to spire, taking up about one third of the shell height, moderately reflected, projecting beyond shell (Fig. 11C, K) and curved back in profile view (Fig. 11D, L). Columellar margin has a large callus and an acute entering fold above. Parietal denticle thick, downward projecting, prominently visible from the umbilical perspective (Fig. 11E, G). Columella two-tiered with two, sinuous, thin, tightly coiled lamellae culminating in the thick parietal denticle (Fig. 12A–D). In dorsal perspective, the columellar lamella slants directly diagonal with its thickest part at the bottom end and projecting moderately from the columella (Fig. 12E–F). Remarkable is the side perspective with aperture facing right (Fig. 12I–J), whereby the columellar lamella takes on a strongly curved tongue-like form directly over the dense, blister-like second lamella of the two-tiered columellar apparatus. The base of the columella spindle is short and obliquely set. The high degree of sinuosity and tight coiling of the two-tiered lamella is remarkable for this species.

###### Differential diagnosis.

Differs from *C. hardiei* sp. n. by its generally more robust and fatter shell, greater degree of whorl convexity and general reduction of distinct striation on the teleoconch. In side view aperture-left profile, the peristome of *C. belizeense* is practically sheer with the convexity of the body whorl whereby in *C. hardiei*, the body whorl projects c. 1/6 beyond the rim of the peristome. The columellar callus on the inner edge of the peristome of *C. belizeense* is much more prominent than in *C. hardiei* as is the remarkably thick and prominent parietal denticle. Internally, by the highly sinuate, tightly coiled two-tiered columellar configuration versus the singular and simple, moderately sinuate lamellar configuration of *C. hardiei*; from *C. floridanum* by the increased convexity of the body whorl giving the impression of a fatter shell, by the decreased concentration of callus in the interior part of the outermost peristome rim causing less an alabaster-like appearance in the side view, aperture-facing-left perspective. Internally, by the highly sinuate, tightly coiled, two-tiered columellar configuration versus the singular and sinuate lamellar configuration of *C. floridanum*; differs from *C. mexicanum* by the more elongate shell and the possession of an additional whorl, by the uneven roundish, flat expanse of the peristome onto the body whorl whereby, in *C. mexicanum*, this feature seems to vary from a straight seam on the surface of the body whorl (Fig. 8A) to a fully-callused expansive peristome with a straight seam as in *C. costaricanum* (Figs 2F, 8I). The parieto-columellar callus is more pronounced in both *C. mexicanum* and *C. costaricanum*. Internally, the highly sinuate, tightly coiled, two-tiered columellar configuration of *C. belizeense* is greatly elaborate (Fig. 12C–D) compared to the less complex, and smoother sinuosity of the lamellar configurations of *C. mexicanum* and *C. costaricanum* (Fig. 9). In *C. mexicanum*, the narrow lamella unfolds mid-way from within the penultimate whorl position into a moderate tongue-like projection (Fig. 9E, G), maintaining a slightly thickened S-shaped sinuous form when turning the shell from dorsal (Fig. 9E–G) to aperture-right perspective (Fig. 9I), whereby for *C. costaricanum*, the lamella is wider at its source in the penultimate whorl, descending smoothly without elaboration. The parietal denticle in *C. belizeense* is stronger and larger than in both *C. mexicanum* and *C. costaricanum*. *Carychium jardineanum* is conchologically distinct from *C. hardiei* in that it is more tapered, more elongated and slender in form, clearly ribbed and bears one additional whorl.

DNA barcode data can clearly delineate *Carychium belizeense* sp. n. from all other North and Central American taxa, demonstrating its lowest K2P genetic distance of 3.9 % to *C. costaricanum* and *C. jardineanum* (Fig. 3), respectively.

###### Etymology.

The new species is named after Belize, the Central American country of origin.

###### Distribution.

Only known from the type locality.

###### Ecology.

Tropical rainforest and karst geology of the eastern slopes of the Mayan Mountain Divide (Dourson and Caldwell 2017).

###### Conservation.

*Carychium belizeense* sp. n. is so far only known from the Bladen Nature Reserve (BNR). Since it was found abundantly and is considered “a fairly common species where it is found living between layers of moist leaves” (Dan Dourson pers. comm.) it is likely not immediately threatened on the highly protected Bladen Nature Reserve. Still, on a global scale, its current distribution is probably limited to the 400-square km comprising the BNR and thus, it constitutes an Endangered narrow range endemic (EN B1) in conjunction with the Guidelines for the IUCN Red List (IUCN Standards and petitions Subcommittee 2014). Since the Bladen Nature Reserve is a major conservation priority in Belize, we are confident *C. belizeense* sp. n. is thriving well because of this protection.

###### Remarks.

The primary upper columellar lamella is incredibly thin, almost like gauze at the line of flexure where it projects away from the columella (Fig. 12A–B). Two scans were necessary to interpret the correct position and fragility of this structure (one slightly damaged in the process). The high extent of sinuosity is remarkable for this species.

In their upcoming publication, Daniel C. Dourson and Ronald S. Caldwell (2017) mention “in the first few whorls, there are minute spiral papillae (beads) which can only be seen under high magnification” (Dan Dourson pers. comm. 2013). In our CT scans, we cannot detect this feature. If pitting on the protoconch is meant here, and only detectable via SEM, this is a common character known throughout the Carychiidae (Burch and Schrader van Devender 1978, Bank and Gittenberger 1985, Jochum 2011) and not unique to this species.

Internally, *Carychium belizeense* is conchologically quite different from all other North American and Central American species hitherto described. In shape and shell robustness, it lies between *C. mexicanum* and *C. costaricanum*. *Carychium jardineanum* is conchologically distinct from *C. belizeanum* in that it is more tapered, elongate and slender in form, clearly ribbed and bears an additional whorl.

##### Carychium zarzaae

Taxon classificationAnimaliaEupulmonataCarychiidae

Jochum & Weigand sp. n.

http://zoobank.org/004706DF-EBA1-4552-B0C6-1A2A966FC970

Figures 13 , 14

Weigand et al., 2013: 3, Fig. 1 49|C13; Seq. ID: BARCA145-12, BARCA146-12, BARCA147-12, BARCA148-12.

###### Material.

Holotype (NMBE 549926/1 ex AJC ex AJC 1902, slightly damaged): Panama, Chiriquí Province, Boquete, path near Boquete; N8.824767, W-82.495833, on moist stick; 12 June 2011; leg. Eugenia Zarza.

**Figure 13.**
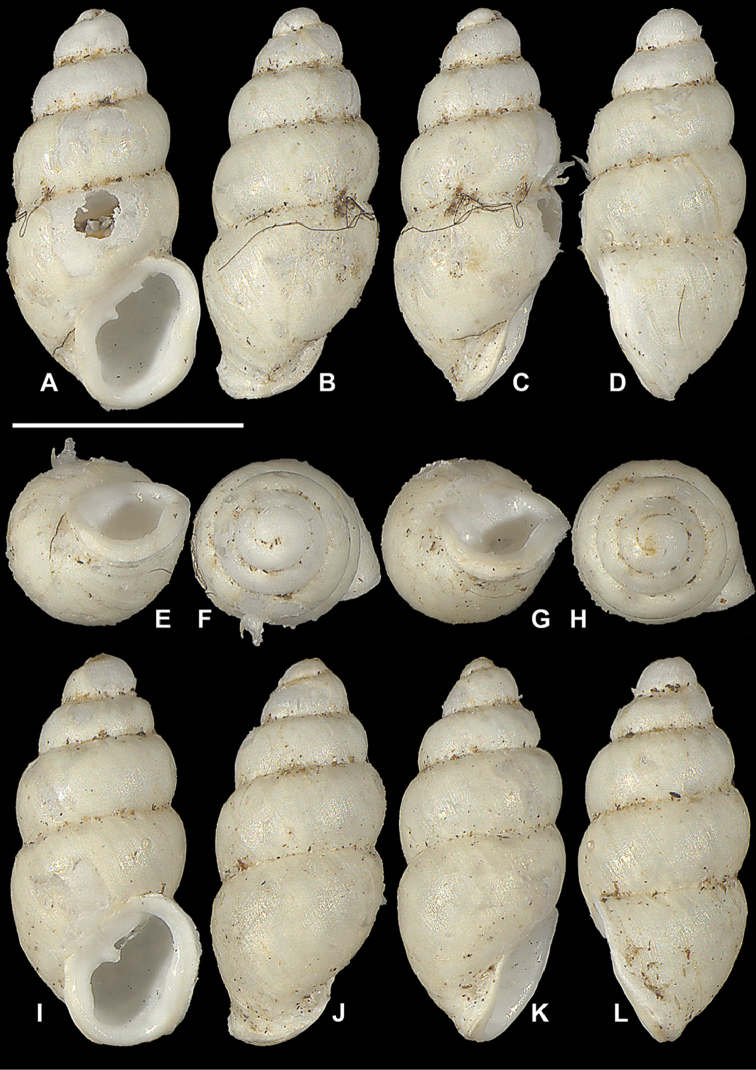
*Carychium zarzaae* sp. n. **A–F** holotype (NMBE 549926/1) **G–L** paratype shell (NMBE 549927/1). Scale bar 1 mm.

**Figure 14.**
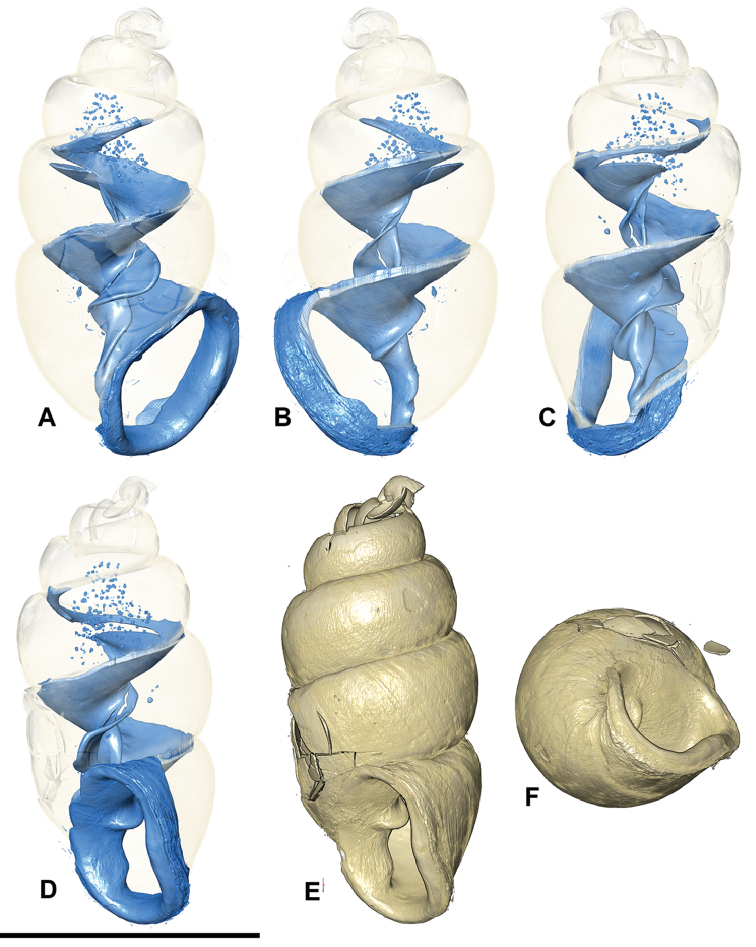
*Carychium zarzaae* sp. n. **A–D** CT scans showing columellar apparatus of paratype (NMBE 549927/1) **D–F** prominent parietal denticle. Scale bar 1 mm.

Paratypes: locus typicus: 1 shell (NMBE 549927/1 ex AJC 1902) slightly damaged (CT-scanned) and 1 shell formerly in formol (SMF 341640 ex AJC 1903); 1 shell formerly in formol (CM 155816 ex AJC 1903); data as the holotype.

###### Diagnosis.

Shell c. 1.72 mm in height, opaque, elongate-pupiform with an entire, elliptically shaped and thickened peristome, with a palatal callus, columellar-basal callus and a moderately sized, parietal denticle.

###### Description.

Measurements are provided in Table 2. Shell minute, elongate-pupiform, opaque, very thin, with about 4.25 convex whorls and a deeply incised suture; with irregular striations or blunt growth lines on the body whorl. Protoconch bulbous. Teleoconch is smooth with glossy sheen. Peristome elliptical and thickly doubled by callus below the palatal bulge (not denticle), only slightly higher than wide, taking up more than one third of the shell height, moderately reflected and curved back at the base (Fig. 13C, I–K). Columellar margin has a parietal-columellar callus. Parietal denticle deeply set and moderately thick, slightly downward projecting and prominently visible from the umbilical perspective (Fig. 14 F). Internally, *C. zarzaae* sp. n. has a very thin sinuous lamella starting at the upper third of the penultimate whorl and forming a moderate tongue-like configuration in the body whorl in dorsal perspective (Fig. 14C). Under the sinuous lamella, the elongated columellar spindle twists smoothly, forming the columellar basal callus at its proximal end (Fig. 14B–C). In dorsal and side-ventral perspectives (Fig. 14C–D), the columellar lamella forms an oblique, elongated S form with its thickest part forming the rim of the distal portion and projecting moderately from the columella (Fig. 14D).

###### Differential diagnosis.

Differs from *C. hardiei* by its very thin shell, rounder whorls and general reduction of marked striation on the teleoconch. In side view aperture-left profile, the body whorl of *C. zarzaae* projects c. 1/8 beyond the rim of the peristome. The shell of *C. zarzaae* is smaller and thinner than *C. costaricanum* and larger and thinner than *C. mexicanum*. The parietal denticle and the parieto-columellar callus on the peristome of *C. zarzaae* are not prominent as in *C. belizeense* (Fig. 11A, E, I) but larger than in *C. hardiei* (Figs 4A, I, 5A, E) and most similar to those in *C. mexicanum* (Puebla) (Fig. 8I–J). The increasing sinuosity of the lamella as it continues into the body whorl in the uppermost part of the penultimate whorl (in the dorsal perspective) of *C. zarzaae* is similar to that of *C. hardiei* and not to *C. mexicanum* nor to *C. costaricanum* (Fig. 9G–H). However, in *C. zarzaae*, the thickness of the lamella and the degree of horizontal lamellar extension is reduced in comparison to this structure in *C. hardiei* (aperture right and left views Fig. 5C–D); differs from *C. mexicanum* by the fatter shell and the reduced parieto-columellar callus seen in some shells from the Puebla *C. mexicanum* population (Fig. 8I–J), by the uneven thickness of callus on the peristome at the palatal mid-section and where it curves back at the basal palatal margin; from *C. costaricanum* by the greatly reduced robustness of the shell and the imbalanced distribution of callus on the peristome. The elongated S-shaped lamella in *C. zarzaae* is most similar to that in *C. mexicanum*. The shell of *C. zarzaae* is homogeneously compact in contrast to the elegantly slender, tapered form of *C. jardineanum*, which also bears nearly six whorls compared to the 4.25 in *Z. zarzaae*.

DNA barcode data can clearly delineate *Carychium zarzaae* sp. n. from all other North and Central American taxa, demonstrating its lowest K2P genetic distance of 4.1 % to *C. jardineanum* (Fig. 3).

###### Etymology.

The new species is named after our Mexican colleague and previous coauthor, Eugenia Zarza, who collected the Panamanian material in Weigand et al. (2013) and subsequently, the Mexican material here. Without her perseverance in the field, we could not have molecularly corroborated Pilsbry’s (1948) morphological investigations, nor had enough comparative material to adequately assess *C. belizeense*. Her field work has opened a new window to Mexican and Central American carychiid diversity and extended the distribution of this genus to its southern-most point in the Western Hemisphere.

###### Distribution.

Only known from the type locality.

###### Ecology.

Tropical rainforest.

###### Conservation.

*Carychium zarzaae* is only known from Boquete. Consequently, and in conjunction with the Guidelines for the IUCN Red List (IUCN Standards and petitions Subcommittee 2014), it is a Critically Endangered narrow range endemic (CR B1) and as such, warrants immediate conservation priority.

###### Remarks.

The very thin shell of *C. zarzaae* contrasts remarkably with the pre-sequence images of the seemingly robust shell of the second Panamanian lineage C12 (Fig. 14), which resembles *C. belizeense* in peristome formation and *C. hardiei* in superficial teleoconch striation. Although somewhat hazy in these images, C12 shows a prominently thickened parieto-columellar callus and a thick parietal denticle in congruence with that in *C. belizeense*. *Carychium zarzaae* is distinct from this joint Panamanian species. The columellar lamella of *C. zarzaae* is incredibly thin, almost like ripped gauze at the line of flexure where it projects away from the columella. In our experience, *C. zarzaae* has the thinnest shell so far encountered within the genus *Carychium*.

**Figure 15.**
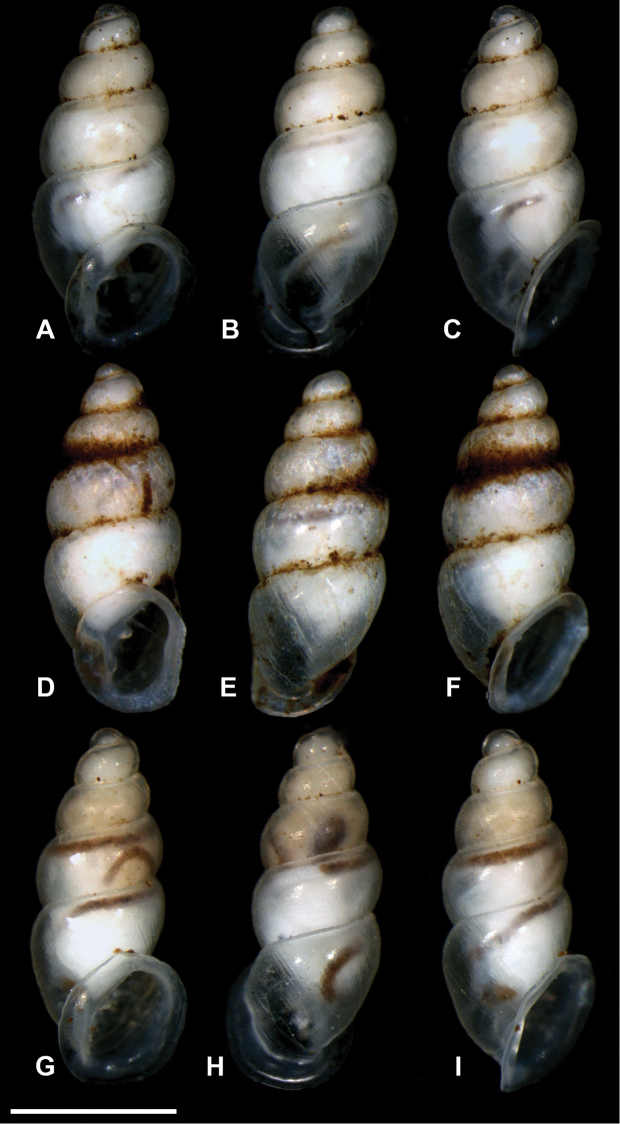
Evolutionary Lineage (EL) C12 (Weigand et al. 2013). **A–I** Panamanian snails before sequence procedures. Scale bar 1 mm.

## Supplementary Material

XML Treatment for Carychium hardiei

XML Treatment for Carychium belizeense

XML Treatment for Carychium zarzaae
